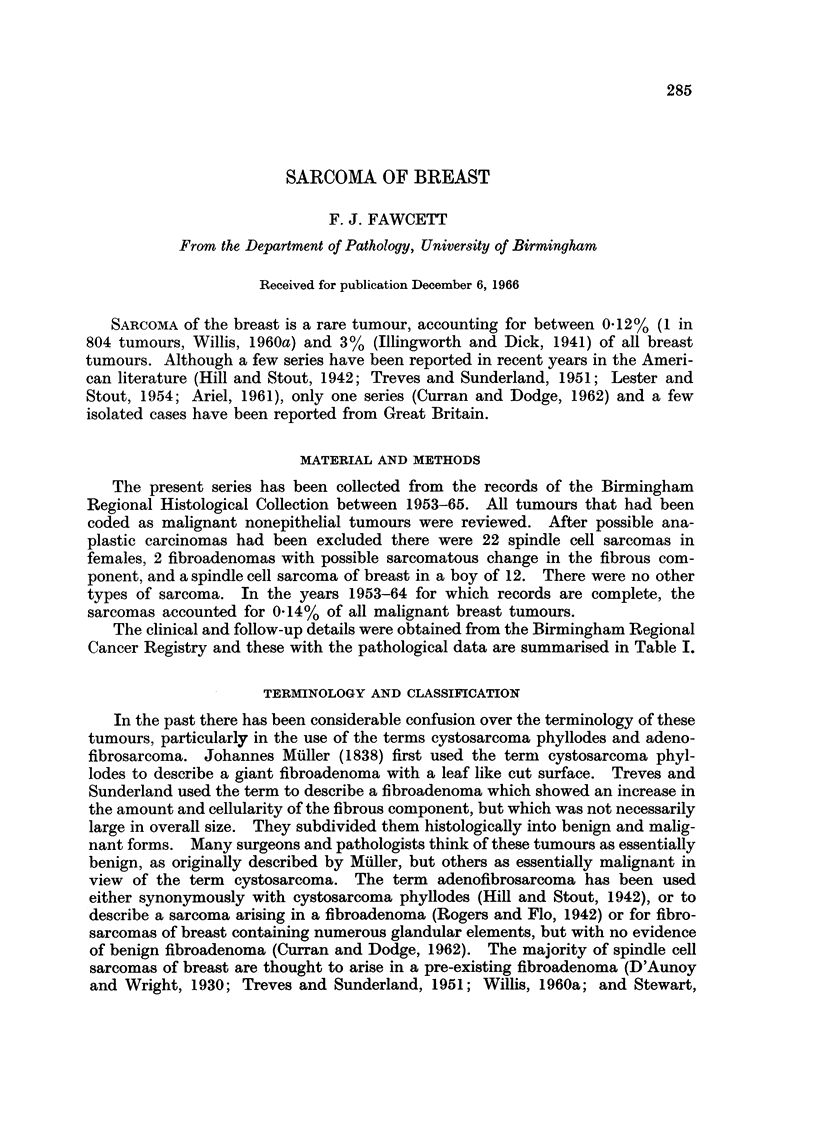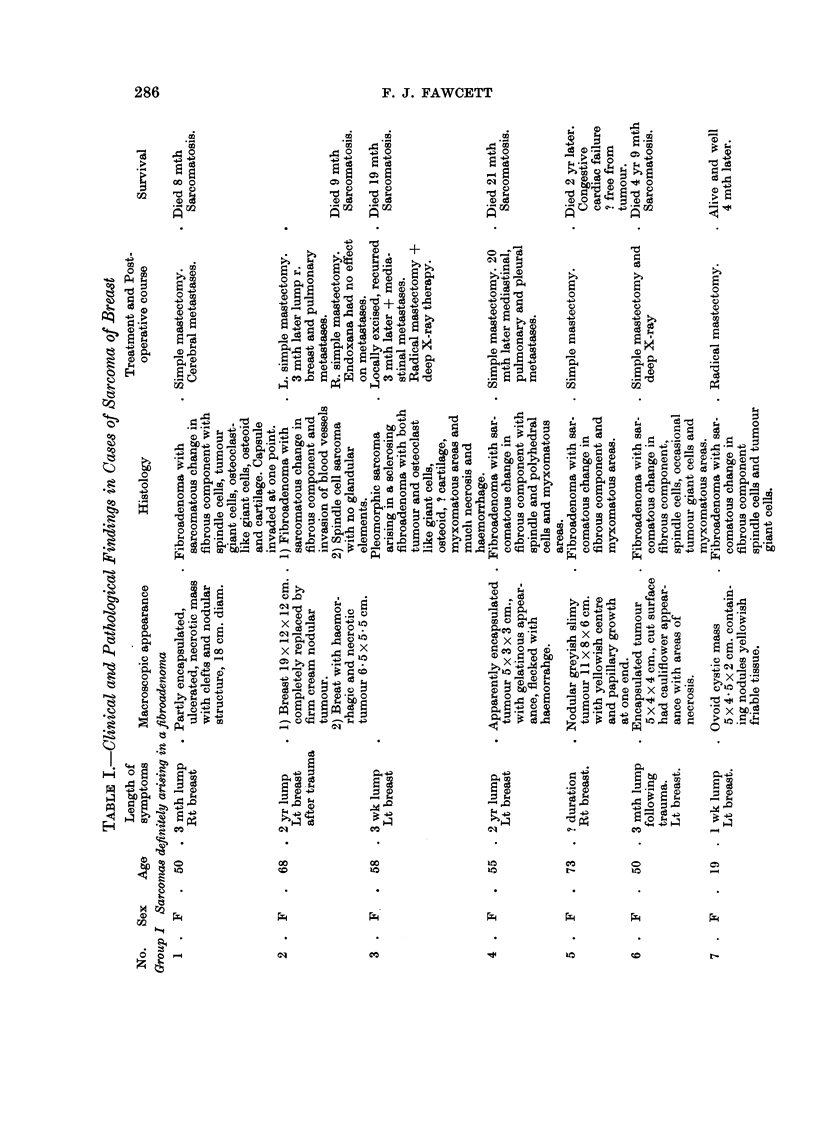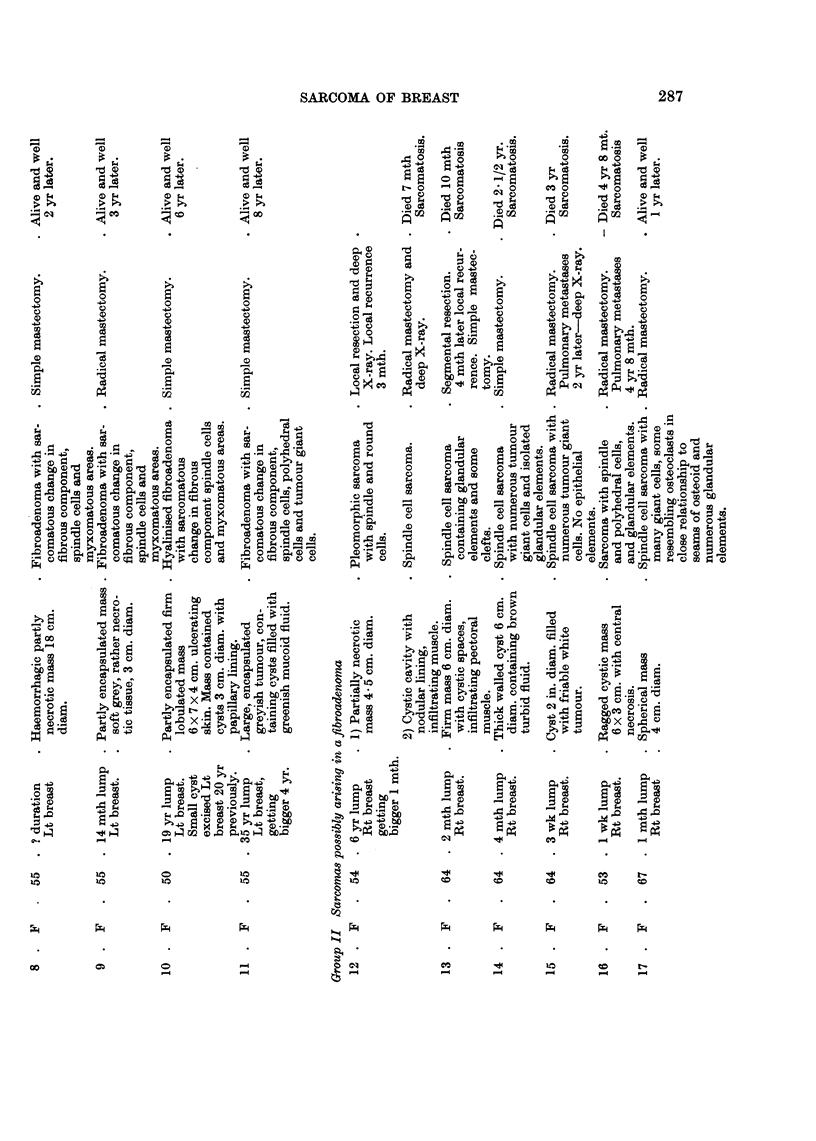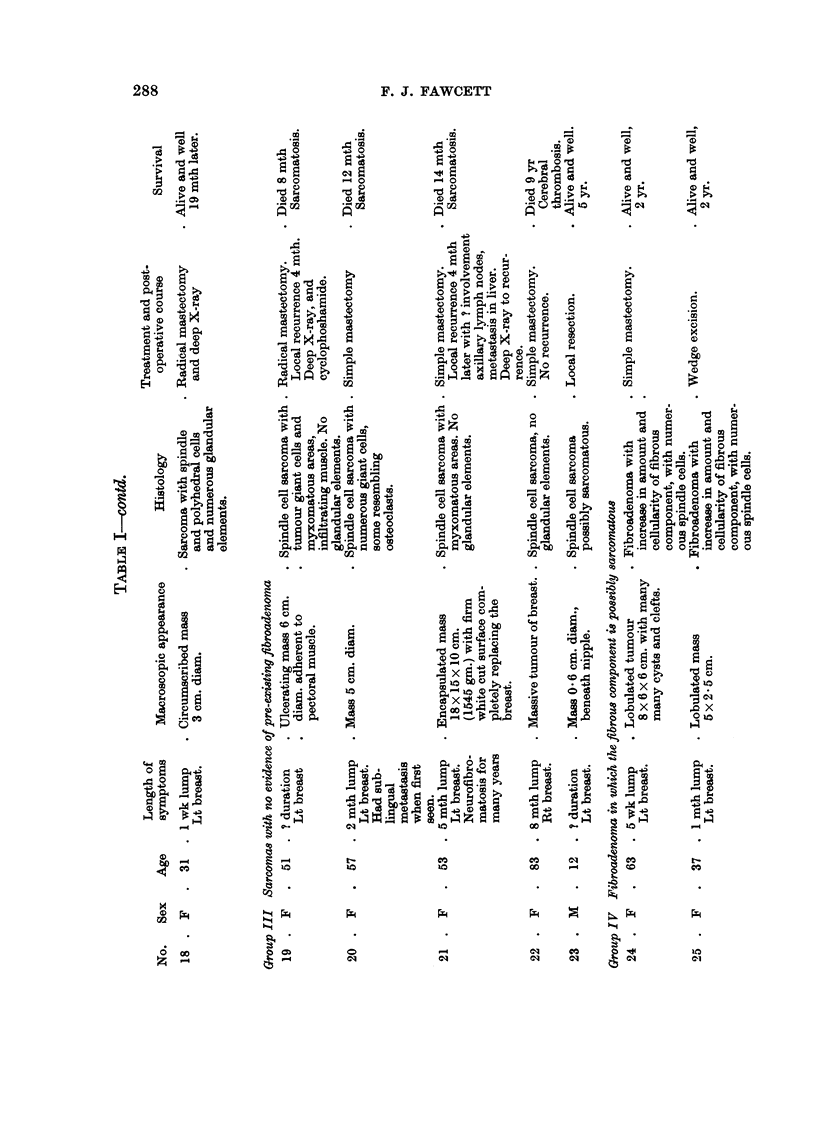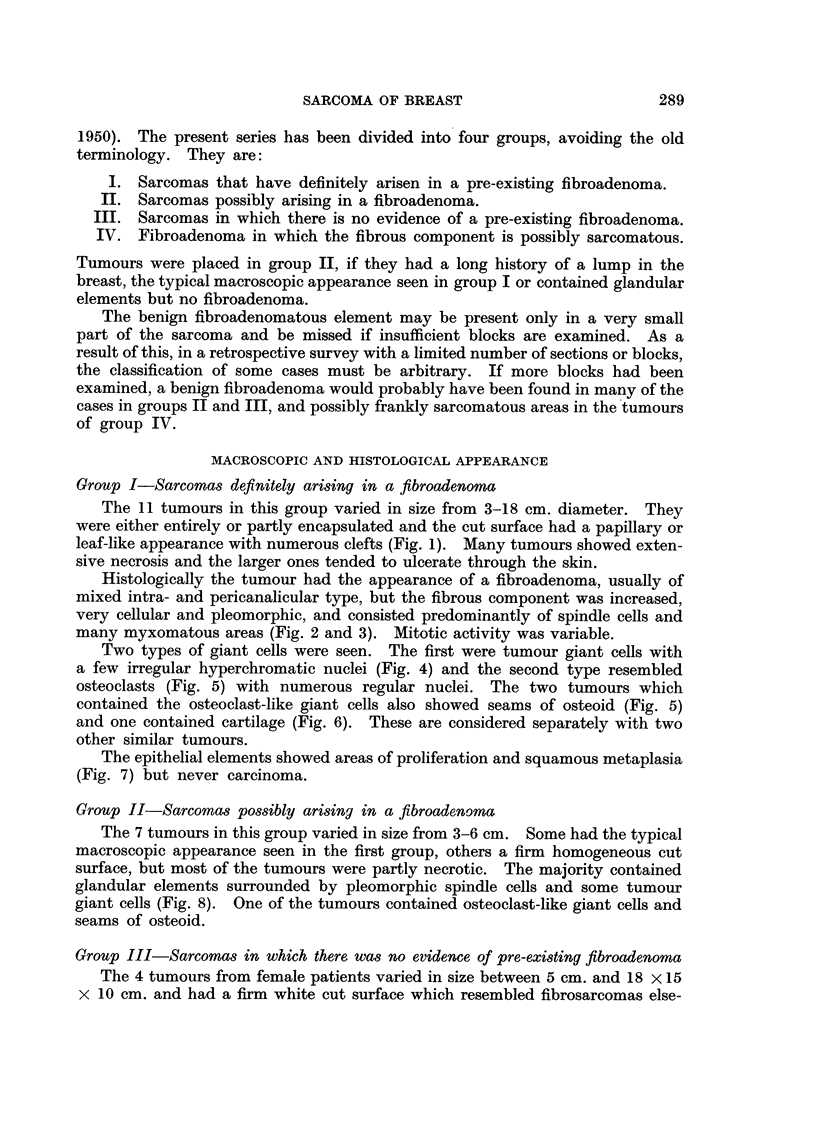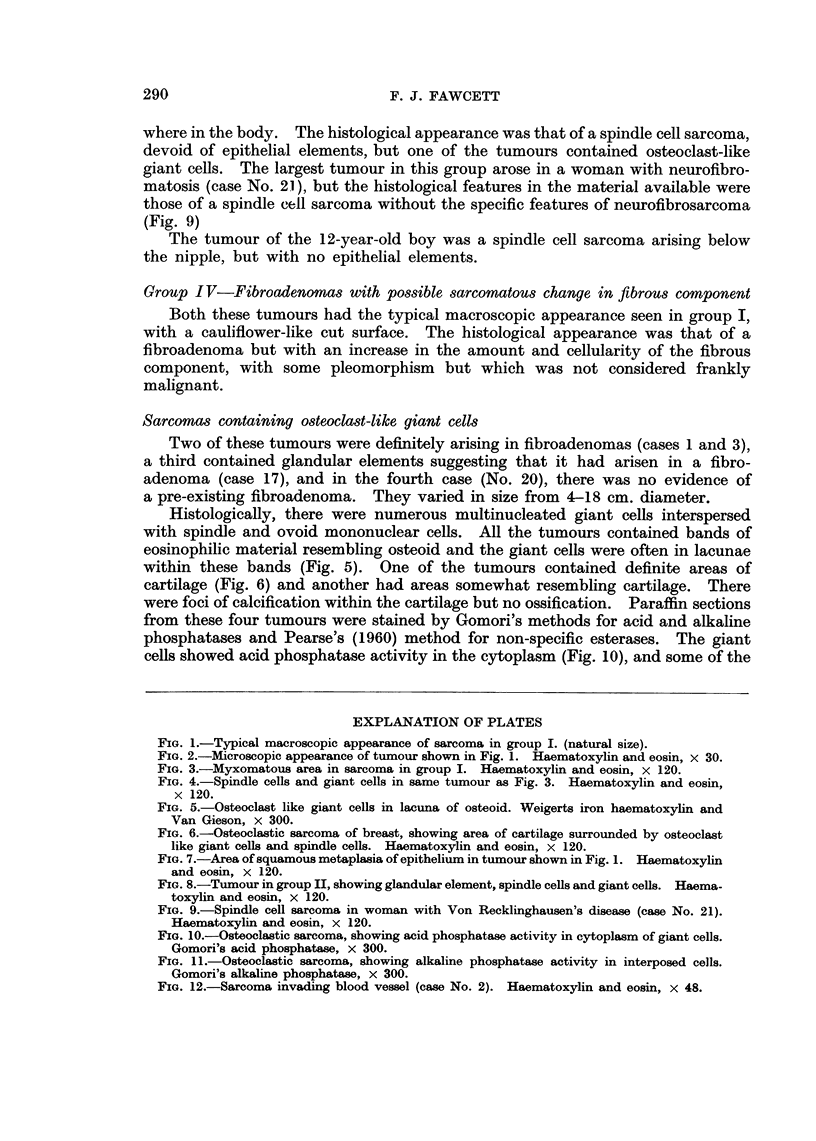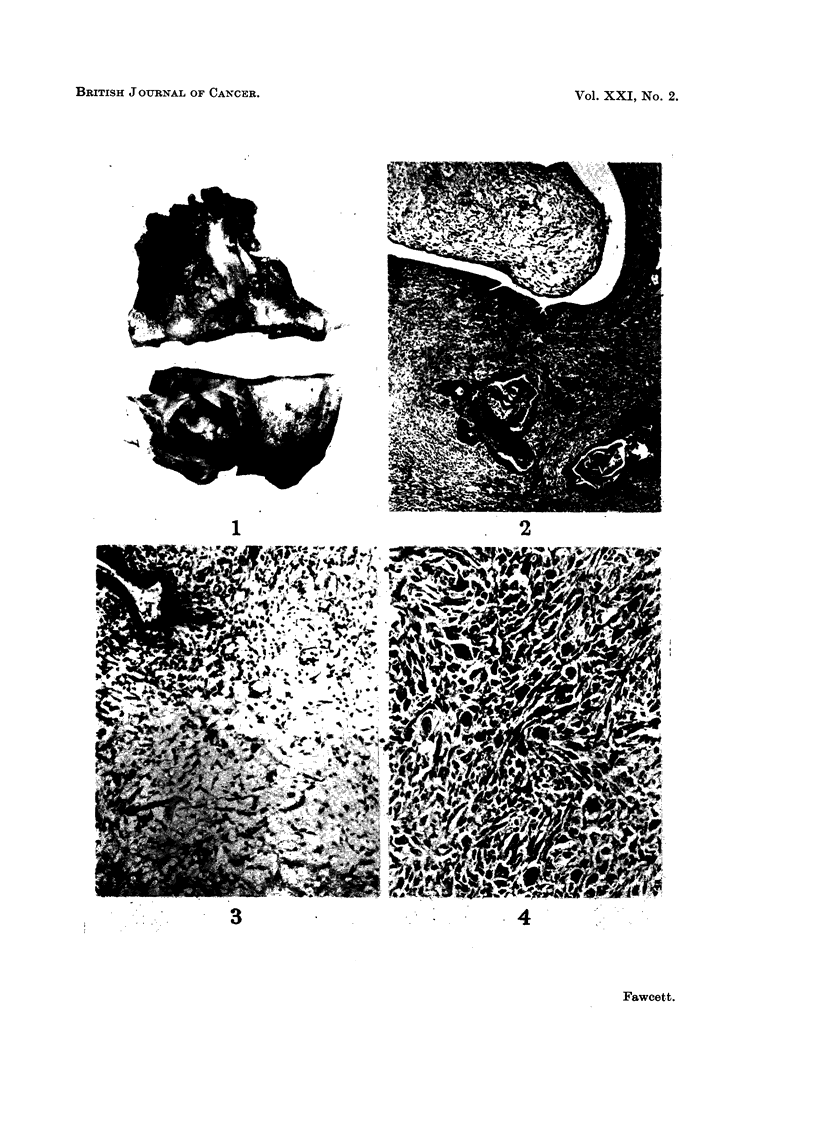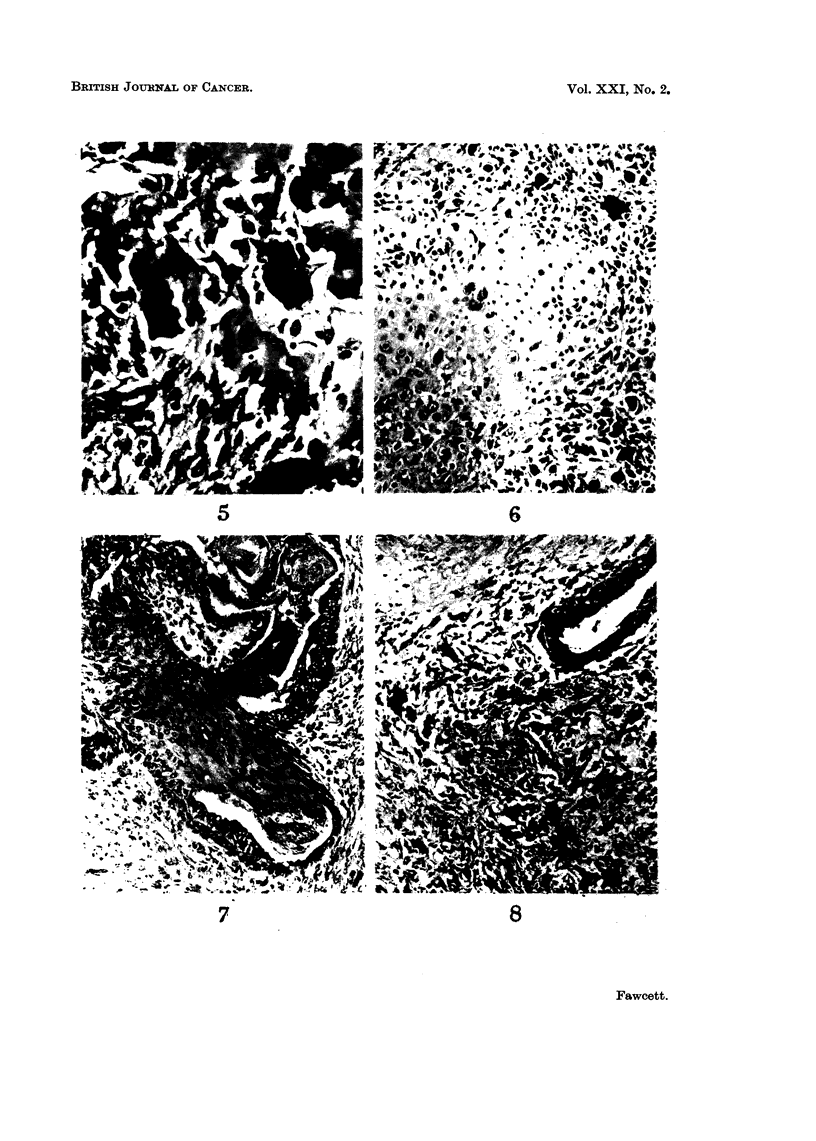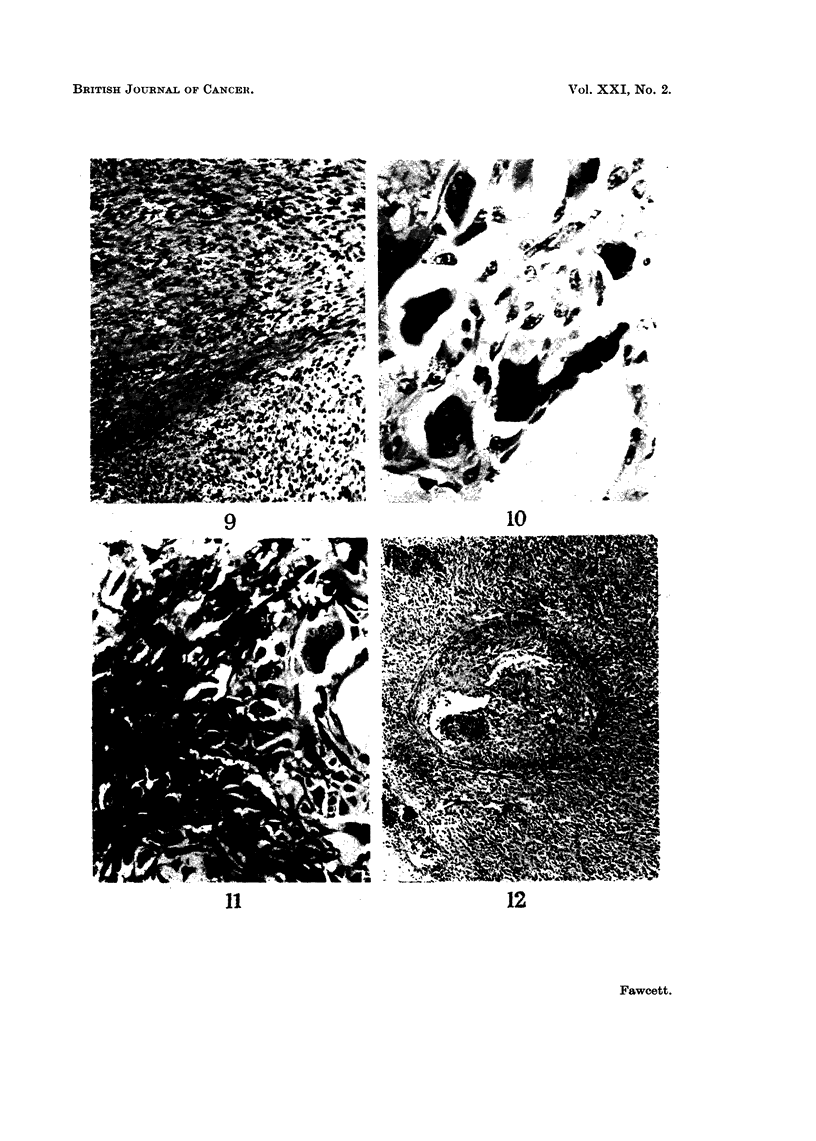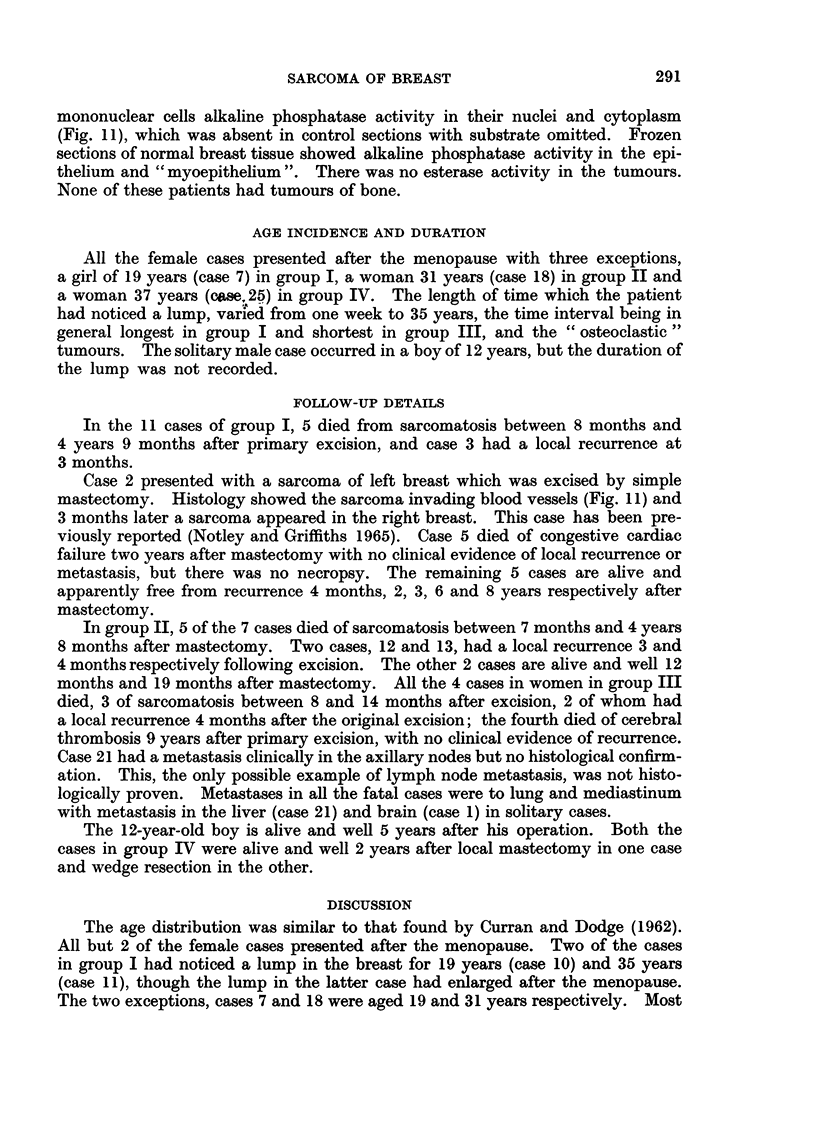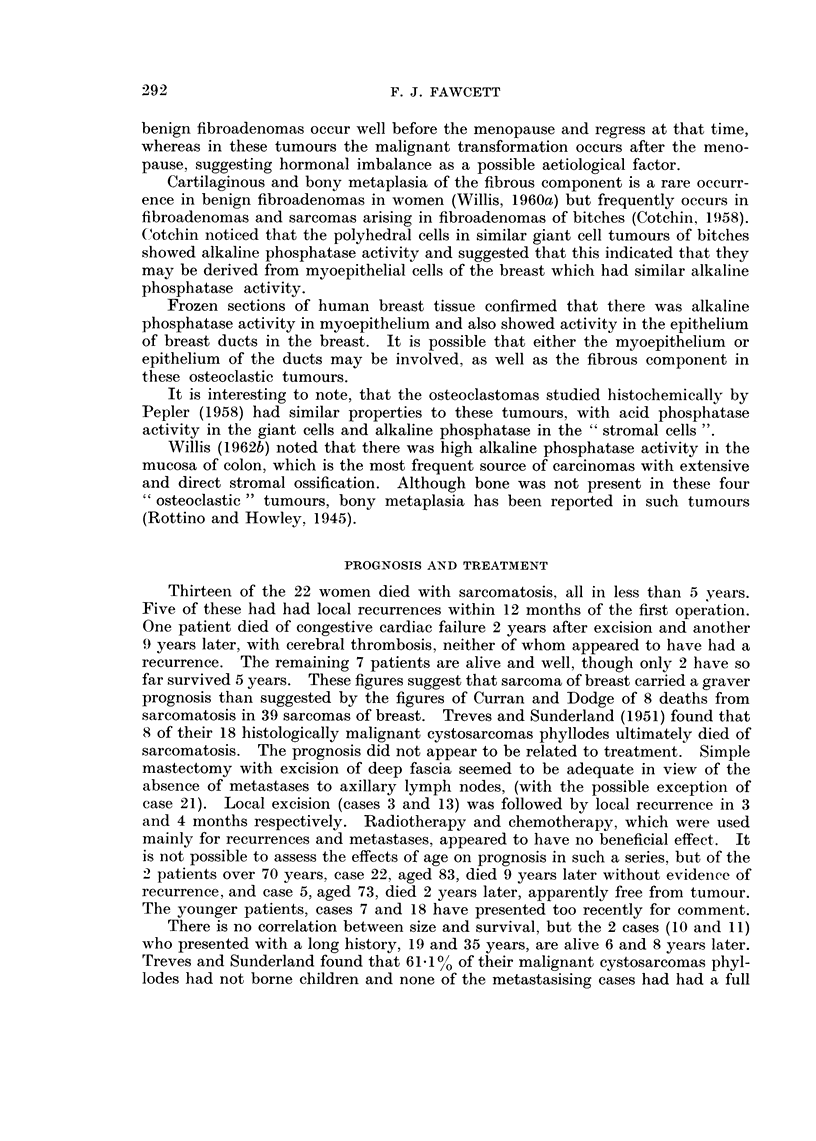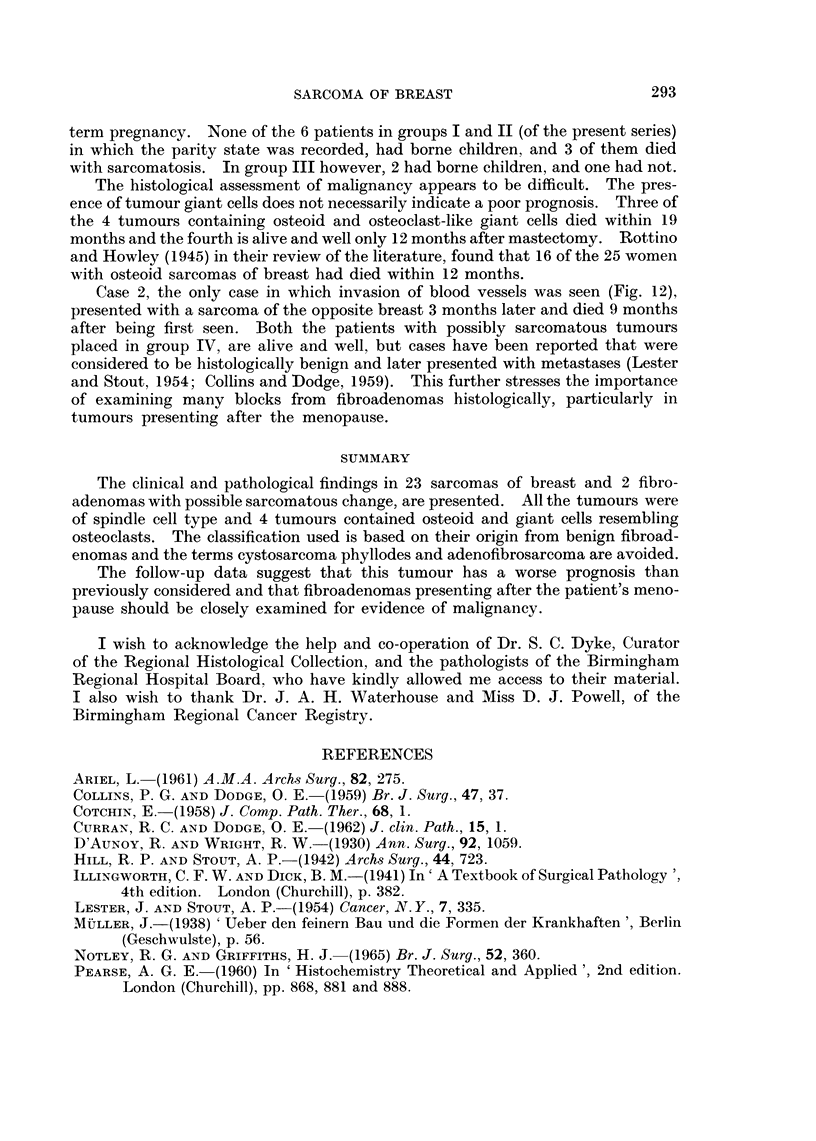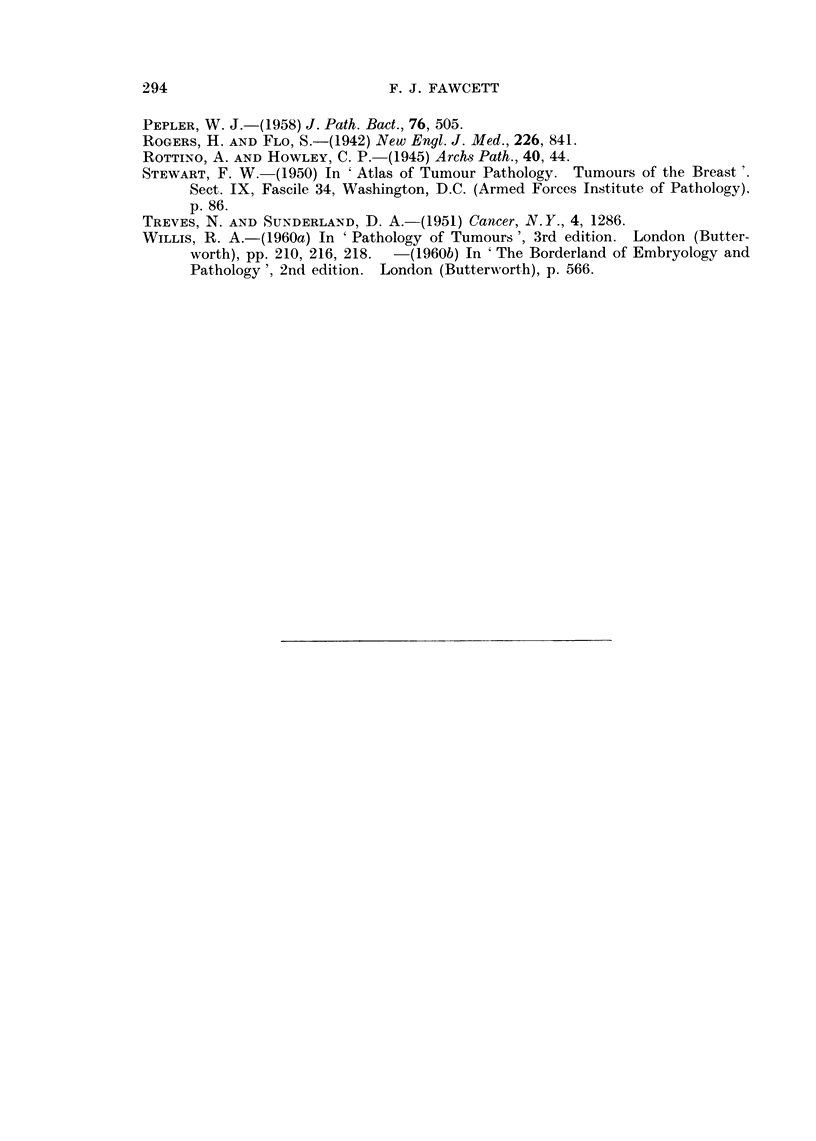# Sarcoma of breast.

**DOI:** 10.1038/bjc.1967.31

**Published:** 1967-06

**Authors:** F. J. Fawcett

## Abstract

**Images:**


### SARCOMA OF BREAST

F. J. FAWCETT

*From the Department of Pathology, University of Birmingham*

Received for publication December 6, 1966

SARCOMA of the breast is a rare tumour, accounting for between 0·12% (1 in 804 tumours, Willis, 1960*a*) and 3% (Illingworth and Dick, 1941) of all breast tumours. Although a few series have been reported in recent years in the American literature (Hill and Stout, 1942; Treves and Sunderland, 1951; Lester and Stout, 1954; Ariel, 1961), only one series (Curran and Dodge, 1962) and a few isolated cases have been reported from Great Britain.

### MATERIAL AND METHODS

The present series has been collected from the records of the Birmingham Regional Histological Collection between 1953–65. All tumours that had been coded as malignant nonepithelial tumours were reviewed. After possible anaplastic carcinomas had been excluded there were 22 spindle cell sarcomas in females, 2 fibroadenomas with possible sarcomatous change in the fibrous component, and a spindle cell sarcoma of breast in a boy of 12. There were no other types of sarcoma. In the years 1953–64 for which records are complete, the sarcomas accounted for 0·14% of all malignant breast tumours.

The clinical and follow-up details were obtained from the Birmingham Regional Cancer Registry and these with the pathological data are summarised in Table I.

### TERMINOLOGY AND CLASSIFICATION

In the past there has been considerable confusion over the terminology of these tumours, particularly in the use of the terms cystosarcoma phyllodes and adenofibrosarcoma. Johannes Müller (1838) first used the term cystosarcoma phyllodes to describe a giant fibroadenoma with a leaf like cut surface. Treves and Sunderland used the term to describe a fibroadenoma which showed an increase in the amount and cellularity of the fibrous component, but which was not necessarily large in overall size. They subdivided them histologically into benign and malignant forms. Many surgeons and pathologists think of these tumours as essentially benign, as originally described by Müller, but others as essentially malignant in view of the term cystosarcoma. The term adenofibrosarcoma has been used either synonymously with cystosarcoma phyllodes (Hill and Stout, 1942), or to describe a sarcoma arising in a fibroadenoma (Rogers and Flo, 1942) or for fibrosarcomas of breast containing numerous glandular elements, but with no evidence of benign fibroadenoma (Curran and Dodge, 1962). The majority of spindle cell sarcomas of breast are thought to arise in a pre-existing fibroadenoma (D'Aunoy and Wright, 1930; Treves and Sunderland, 1951; Willis, 1960a; and Stewart,

TABLE I.—*Clinical and Pathological Findings in Cases of Sarcoma of Breast*

| No. | Sex | Age | Length of symptoms | Macroscopic appearance | Histology | Treatment and Post-operative course | Survival |
|---|---|---|---|---|---|---|---|
| *Group I* | *Sarcomas definitely arising in a fibroadenoma* | | | | | | |
| 1 | F | 50 | 3 mth lump Rt breast | Partly encapsulated, ulcerated, necrotic mass with clefts and nodular structure, 18 cm. diam. | Fibroadenoma with sarcomatous change in fibrous component with spindle cells, tumour giant cells, osteoclast-like giant cells, osteoid and cartilage. Capsule invaded at one point. | Simple mastectomy. Cerebral metastases. | Died 8 mth Sarcomatosis. |
| 2 | F | 68 | 2 yr lump Lt breast after trauma | 1) Breast 19 × 12 × 12 cm. completely replaced by firm cream nodular tumour. 2) Breat with haemorrhagic and necrotic tumour 6·5 × 5·5 cm. | 1) Fibroadenoma with sarcomatous change in fibrous component and invasion of blood vessels 2) Spindle cell sarcoma with no glandular elements. | L. simple mastectomy. 3 mth later lump r. breast and pulmonary metastases. R. simple mastectomy. Endoxana had no effect on metastases. | Died 9 mth Sarcomatosis. |
| 3 | F | 58 | 3 wk lump Lt breast | | Pleomorphic sarcoma arising in a sclerosing fibroadenoma with both tumour and osteoclast like giant cells, osteoid, ? cartilage, myxomatous areas and much necrosis and haemorrhage. | Locally excised, recurred 3 mth later + mediastinal metastases. Radical mastectomy + deep X-ray therapy. | Died 19 mth Sarcomatosis. |
| 4 | F | 55 | 2 yr lump Lt breast | Apparently encapsulated tumour 5 × 3 × 3 cm., with gelatinous appearance, flecked with haemorrahge. | Fibroadenoma with sarcomatous change in fibrous component with spindle and polyhedral cells and myxomatous areas. | Simple mastectomy. 20 mth later mediastinal, pulmonary and pleural metastases. | Died 21 mth Sarcomatosis. |
| 5 | F | 73 | ? duration Rt breast. | Nodular greyish slimy tumour 11 × 8 × 6 cm. with yellowish centre and papillary growth at one end. | Fibroadenoma with sarcomatous change in fibrous component and myxomatous areas. | Simple mastectomy. | Died 2 yr later. Congestive cardiac failure ? free from tumour. |
| 6 | F | 50 | 3 mth lump following trauma. Lt breast. | Encapsulated tumour 5 × 4 × 4 cm., cut surface had cauliflower appearance with areas of necrosis. | Fibroadenoma with sarcomatous change in fibrous component, spindle cells, occasional tumour giant cells and myxomatous areas. | Simple mastectomy and deep X-ray | Died 4 yr 9 mth Sarcomatosis. |
| 7 | F | 19 | 1 wk lump Lt breast. | Ovoid cystic mass 5 × 4·5 × 2 cm. containing nodules yellowish friable tissue. | Fibroadenoma with sarcomatous change in fibrous component spindle cells and tumour giant cells. | Radical mastectomy. | Alive and well 4 mth later. |

| | | | | | | | |
|---|---|---|---|---|---|---|---|
| 8 | F | 55 | ? duration Lt breast | Haemorrhagic partly necrotic mass 18 cm. diam. | Fibroadenoma with sarcomatous change in fibrous component, spindle cells and myxomatous areas. | Simple mastectomy. | Alive and well 2 yr later. |
| 9 | F | 55 | 14 mth lump Lt breast. | Partly encapsulated mass soft grey, rather necrotic tissue, 3 cm. diam. | Fibroadenoma with sarcomatous change in fibrous component, spindle cells and myxomatous areas. | Radical mastectomy. | Alive and well 3 yr later. |
| 10 | F | 50 | 19 yr lump Lt breast. Small cyst excised Lt breast 20 yr previously. | Partly encapsulated firm lobulated mass 6 × 7 × 4 cm. ulcerating skin. Mass contained cysts 3 cm. diam. with papillary lining. | Hyalinised fibroadenoma with sarcomatous change in fibrous component spindle cells and myxomatous areas. | Simple mastectomy. | Alive and well 6 yr later. |
| 11 | F | 55 | 35 yr lump Lt breast, getting bigger 4 yr. | Large, encapsulated greyish tumour, containing cysts filled with greenish mucoid fluid. | Fibroadenoma with sarcomatous change in fibrous component, spindle cells, polyhedral cells and tumour giant cells. | Simple mastectomy. | Alive and well 8 yr later. |
| *Group II* | *Sarcomas possibly arising in a fibroadenoma* | | | | | | |
| 12 | F | 54 | 6 yr lump Rt breast getting bigger 1 mth. | 1) Partially necrotic mass 4·5 cm. diam. | Pleomorphic sarcoma with spindle and round cells. | Local resection and deep X-ray. Local recurrence 3 mth. | |
| | | | | 2) Cystic cavity with nodular lining, infiltrating muscle. | Spindle cell sarcoma. | Radical mastectomy and deep X-ray. | Died 7 mth Sarcomatosis. |
| 13 | F | 64 | 2 mth lump Rt breast. | Firm mass 6 cm. diam. with cystic spaces, infiltrating pectoral muscle. | Spindle cell sarcoma containing glandular elements and some clefts. | Segmental resection. 4 mth later local recurrence. Simple mastectomy. | Died 10 mth Sarcomatosis |
| 14 | F | 64 | 4 mth lump Rt breast. | Thick walled cyst 6 cm. diam. containing brown turbid fluid. | Spindle cell sarcoma with numerous tumour giant cells and isolated glandular elements. | Simple mastectomy. | Died 2·1/2 yr. Sarcomatosis. |
| 15 | F | 64 | 3 wk lump Rt breast. | Cyst 2 in. diam. filled with friable white tumour. | Spindle cell sarcoma with numerous tumour giant cells. No epithelial elements. | Radical mastectomy. Pulmonary metastases 2 yr later—deep X-ray. | Died 3 yr Sarcomatosis. |
| 16 | F | 53 | 1 wk lump Rt breast. | Ragged cystic mass 6 × 3 cm. with central necrosis. | Sarcoma with spindle and polyhedral cells, and glandular elements. | Radical mastectomy. Pulmonary metastases 4 yr 8 mth. | Died 4 yr 8 mt. Sarcomatosis |
| 17 | F | 67 | 1 mth lump Rt breast | Spherical mass 4 cm. diam. | Spindle cell sarcoma with many giant cells, some resembling osteoclasts in close relationship to seams of osteoid and numerous glandular elements. | Radical mastectomy. | Alive and well 1 yr later. |

TABLE I—*contd.*

| No. | Sex | Age | Length of symptoms | Macroscopic appearance | Histology | Treatment and post-operative course | Survival |
|---|---|---|---|---|---|---|---|
| 18 | F | 31 | 1 wk lump Lt breast. | Circumscribed mass 3 cm. diam. | Sarcoma with spindle and polyhedral cells and numerous glandular elements. | Radical mastectomy and deep X-ray | Alive and well 19 mth later. |
| *Group III* | *Sarcomas with no evidence of pre-existing fibroadenoma* | | | | | | |
| 19 | F | 51 | ? duration Lt breast | Ulcerating mass 6 cm. diam. adherent to pectoral muscle. | Spindle cell sarcoma with tumour giant cells and myxomatous areas, infiltrating muscle. No glandular elements. | Radical mastectomy. Local recurrence 4 mth. Deep X-ray, and cyclophoshamide. | Died 8 mth Sarcomatosis. |
| 20 | F | 57 | 2 mth lump Lt breast. Had sub-lingual metastasis when first seen. | Mass 5 cm. diam. | Spindle cell sarcoma with numerous giant cells, some resembling osteoclasts. | Simple mastectomy | Died 12 mth Sarcomatosis. |
| 21 | F | 53 | 5 mth lump Lt breast. Neurofibromatosis for many years | Encapsulated mass 18 × 15 × 10 cm. (1545 gm.) with firm white cut surface completely replacing the breast. | Spindle cell sarcoma with myxomatous areas. No glandular elements. | Simple mastectomy. Local recurrence 4 mth later with ? involvement axillary lymph nodes, metastasis in liver. Deep X-ray to recurrence. | Died 14 mth Sarcomatosis. |
| 22 | F | 83 | 8 mth lump Rt breast. | Massive tumour of breast. | Spindle cell sarcoma, no glandular elements. | Simple mastectomy. No recurrence. | Died 9 yr Cerebral thrombosis. |
| 23 | M | 12 | ? duration Lt breast. | Mass 0·6 cm. diam., beneath nipple. | Spindle cell sarcoma possibly sarcomatous. | Local resection. | Alive and well. 5 yr. |
| *Group IV* | *Fibroadenoma in which the fibrous component is possibly sarcomatous* | | | | | | |
| 24 | F | 63 | 5 wk lump Lt breast. | Lobulated tumour 8 × 6 × 6 cm. with many many cysts and clefts. | Fibroadenoma with increase in amount and cellularity of fibrous component, with numerous spindle cells. | Simple mastectomy. | Alive and well, 2 yr. |
| 25 | F | 37 | 1 mth lump Lt breast. | Lobulated mass 5 × 2·5 cm. | Fibroadenoma with increase in amount and cellularity of fibrous component, with numerous spindle cells. | Wedge excision. | Alive and well, 2 yr. |

1950). The present series has been divided into four groups, avoiding the old terminology. They are:

I. Sarcomas that have definitely arisen in a pre-existing fibroadenoma.
II. Sarcomas possibly arising in a fibroadenoma.
III. Sarcomas in which there is no evidence of a pre-existing fibroadenoma.
IV. Fibroadenoma in which the fibrous component is possibly sarcomatous.

Tumours were placed in group II, if they had a long history of a lump in the breast, the typical macroscopic appearance seen in group I or contained glandular elements but no fibroadenoma.

The benign fibroadenomatous element may be present only in a very small part of the sarcoma and be missed if insufficient blocks are examined. As a result of this, in a retrospective survey with a limited number of sections or blocks, the classification of some cases must be arbitrary. If more blocks had been examined, a benign fibroadenoma would probably have been found in many of the cases in groups II and III, and possibly frankly sarcomatous areas in the tumours of group IV.

### MACROSCOPIC AND HISTOLOGICAL APPEARANCE

*Group I—Sarcomas definitely arising in a fibroadenoma*

The 11 tumours in this group varied in size from 3–18 cm. diameter. They were either entirely or partly encapsulated and the cut surface had a papillary or leaf-like appearance with numerous clefts (Fig. 1). Many tumours showed extensive necrosis and the larger ones tended to ulcerate through the skin.

Histologically the tumour had the appearance of a fibroadenoma, usually of mixed intra- and pericanalicular type, but the fibrous component was increased, very cellular and pleomorphic, and consisted predominantly of spindle cells and many myxomatous areas (Fig. 2 and 3). Mitotic activity was variable.

Two types of giant cells were seen. The first were tumour giant cells with a few irregular hyperchromatic nuclei (Fig. 4) and the second type resembled osteoclasts (Fig. 5) with numerous regular nuclei. The two tumours which contained the osteoclast-like giant cells also showed seams of osteoid (Fig. 5) and one contained cartilage (Fig. 6). These are considered separately with two other similar tumours.

The epithelial elements showed areas of proliferation and squamous metaplasia (Fig. 7) but never carcinoma.

*Group II—Sarcomas possibly arising in a fibroadenoma*

The 7 tumours in this group varied in size from 3–6 cm. Some had the typical macroscopic appearance seen in the first group, others a firm homogeneous cut surface, but most of the tumours were partly necrotic. The majority contained glandular elements surrounded by pleomorphic spindle cells and some tumour giant cells (Fig. 8). One of the tumours contained osteoclast-like giant cells and seams of osteoid.

*Group III—Sarcomas in which there was no evidence of pre-existing fibroadenoma*

The 4 tumours from female patients varied in size between 5 cm. and 18 $\times$15 $\times$ 10 cm. and had a firm white cut surface which resembled fibrosarcomas elsewhere in the body. The histological appearance was that of a spindle cell sarcoma, devoid of epithelial elements, but one of the tumours contained osteoclast-like giant cells. The largest tumour in this group arose in a woman with neurofibromatosis (case No. 21), but the histological features in the material available were those of a spindle cell sarcoma without the specific features of neurofibrosarcoma (Fig. 9)

The tumour of the 12-year-old boy was a spindle cell sarcoma arising below the nipple, but with no epithelial elements.

*Group IV—Fibroadenomas with possible sarcomatous change in fibrous component*

Both these tumours had the typical macroscopic appearance seen in group I, with a cauliflower-like cut surface. The histological appearance was that of a fibroadenoma but with an increase in the amount and cellularity of the fibrous component, with some pleomorphism but which was not considered frankly malignant.

*Sarcomas containing osteoclast-like giant cells*

Two of these tumours were definitely arising in fibroadenomas (cases 1 and 3), a third contained glandular elements suggesting that it had arisen in a fibroadenoma (case 17), and in the fourth case (No. 20), there was no evidence of a pre-existing fibroadenoma. They varied in size from 4–18 cm. diameter.

Histologically, there were numerous multinucleated giant cells interspersed with spindle and ovoid mononuclear cells. All the tumours contained bands of eosinophilic material resembling osteoid and the giant cells were often in lacunae within these bands (Fig. 5). One of the tumours contained definite areas of cartilage (Fig. 6) and another had areas somewhat resembling cartilage. There were foci of calcification within the cartilage but no ossification. Paraffin sections from these four tumours were stained by Gomori's methods for acid and alkaline phosphatases and Pearse's (1960) method for non-specific esterases. The giant cells showed acid phosphatase activity in the cytoplasm (Fig. 10), and some of the

---

EXPLANATION OF PLATES

FIG. 1.—Typical macroscopic appearance of sarcoma in group I. (natural size).
FIG. 2.—Microscopic appearance of tumour shown in Fig. 1. Haematoxylin and eosin, × 30.
FIG. 3.—Myxomatous area in sarcoma in group I. Haematoxylin and eosin, × 120.
FIG. 4.—Spindle cells and giant cells in same tumour as Fig. 3. Haematoxylin and eosin, × 120.
FIG. 5.—Osteoclast like giant cells in lacuna of osteoid. Weigerts iron haematoxylin and Van Gieson, × 300.
FIG. 6.—Osteoclastic sarcoma of breast, showing area of cartilage surrounded by osteoclast like giant cells and spindle cells. Haematoxylin and eosin, × 120.
FIG. 7.—Area of squamous metaplasia of epithelium in tumour shown in Fig. 1. Haematoxylin and eosin, × 120.
FIG. 8.—Tumour in group II, showing glandular element, spindle cells and giant cells. Haematoxylin and eosin, × 120.
FIG. 9.—Spindle cell sarcoma in woman with Von Recklinghausen's disease (case No. 21). Haematoxylin and eosin, × 120.
FIG. 10.—Osteoclastic sarcoma, showing acid phosphatase activity in cytoplasm of giant cells. Gomori's acid phosphatase, × 300.
FIG. 11.—Osteoclastic sarcoma, showing alkaline phosphatase activity in interposed cells. Gomori's alkaline phosphatase, × 300.
FIG. 12.—Sarcoma invading blood vessel (case No. 2). Haematoxylin and eosin, × 48.

1

2

3

4

Fawcett.

5

6

7

8

Fawcett.

9

10

11

12

mononuclear cells alkaline phosphatase activity in their nuclei and cytoplasm (Fig. 11), which was absent in control sections with substrate omitted. Frozen sections of normal breast tissue showed alkaline phosphatase activity in the epithelium and "myoepithelium". There was no esterase activity in the tumours. None of these patients had tumours of bone.

### AGE INCIDENCE AND DURATION

All the female cases presented after the menopause with three exceptions, a girl of 19 years (case 7) in group I, a woman 31 years (case 18) in group II and a woman 37 years (case 25) in group IV. The length of time which the patient had noticed a lump, varied from one week to 35 years, the time interval being in general longest in group I and shortest in group III, and the "osteoclastic" tumours. The solitary male case occurred in a boy of 12 years, but the duration of the lump was not recorded.

### FOLLOW-UP DETAILS

In the 11 cases of group I, 5 died from sarcomatosis between 8 months and 4 years 9 months after primary excision, and case 3 had a local recurrence at 3 months.

Case 2 presented with a sarcoma of left breast which was excised by simple mastectomy. Histology showed the sarcoma invading blood vessels (Fig. 11) and 3 months later a sarcoma appeared in the right breast. This case has been previously reported (Notley and Griffiths 1965). Case 5 died of congestive cardiac failure two years after mastectomy with no clinical evidence of local recurrence or metastasis, but there was no necropsy. The remaining 5 cases are alive and apparently free from recurrence 4 months, 2, 3, 6 and 8 years respectively after mastectomy.

In group II, 5 of the 7 cases died of sarcomatosis between 7 months and 4 years 8 months after mastectomy. Two cases, 12 and 13, had a local recurrence 3 and 4 months respectively following excision. The other 2 cases are alive and well 12 months and 19 months after mastectomy. All the 4 cases in women in group III died, 3 of sarcomatosis between 8 and 14 months after excision, 2 of whom had a local recurrence 4 months after the original excision; the fourth died of cerebral thrombosis 9 years after primary excision, with no clinical evidence of recurrence. Case 21 had a metastasis clinically in the axillary nodes but no histological confirmation. This, the only possible example of lymph node metastasis, was not histologically proven. Metastases in all the fatal cases were to lung and mediastinum with metastasis in the liver (case 21) and brain (case 1) in solitary cases.

The 12-year-old boy is alive and well 5 years after his operation. Both the cases in group IV were alive and well 2 years after local mastectomy in one case and wedge resection in the other.

### DISCUSSION

The age distribution was similar to that found by Curran and Dodge (1962). All but 2 of the female cases presented after the menopause. Two of the cases in group I had noticed a lump in the breast for 19 years (case 10) and 35 years (case 11), though the lump in the latter case had enlarged after the menopause. The two exceptions, cases 7 and 18 were aged 19 and 31 years respectively. Most benign fibroadenomas occur well before the menopause and regress at that time, whereas in these tumours the malignant transformation occurs after the menopause, suggesting hormonal imbalance as a possible aetiological factor.

Cartilaginous and bony metaplasia of the fibrous component is a rare occurrence in benign fibroadenomas in women (Willis, 1960*a*) but frequently occurs in fibroadenomas and sarcomas arising in fibroadenomas of bitches (Cotchin, 1958). Cotchin noticed that the polyhedral cells in similar giant cell tumours of bitches showed alkaline phosphatase activity and suggested that this indicated that they may be derived from myoepithelial cells of the breast which had similar alkaline phosphatase activity.

Frozen sections of human breast tissue confirmed that there was alkaline phosphatase activity in myoepithelium and also showed activity in the epithelium of breast ducts in the breast. It is possible that either the myoepithelium or epithelium of the ducts may be involved, as well as the fibrous component in these osteoclastic tumours.

It is interesting to note, that the osteoclastomas studied histochemically by Pepler (1958) had similar properties to these tumours, with acid phosphatase activity in the giant cells and alkaline phosphatase in the " stromal cells ".

Willis (1962*b*) noted that there was high alkaline phosphatase activity in the mucosa of colon, which is the most frequent source of carcinomas with extensive and direct stromal ossification. Although bone was not present in these four " osteoclastic " tumours, bony metaplasia has been reported in such tumours (Rottino and Howley, 1945).

### PROGNOSIS AND TREATMENT

Thirteen of the 22 women died with sarcomatosis, all in less than 5 years. Five of these had had local recurrences within 12 months of the first operation. One patient died of congestive cardiac failure 2 years after excision and another 9 years later, with cerebral thrombosis, neither of whom appeared to have had a recurrence. The remaining 7 patients are alive and well, though only 2 have so far survived 5 years. These figures suggest that sarcoma of breast carried a graver prognosis than suggested by the figures of Curran and Dodge of 8 deaths from sarcomatosis in 39 sarcomas of breast. Treves and Sunderland (1951) found that 8 of their 18 histologically malignant cystosarcomas phyllodes ultimately died of sarcomatosis. The prognosis did not appear to be related to treatment. Simple mastectomy with excision of deep fascia seemed to be adequate in view of the absence of metastases to axillary lymph nodes, (with the possible exception of case 21). Local excision (cases 3 and 13) was followed by local recurrence in 3 and 4 months respectively. Radiotherapy and chemotherapy, which were used mainly for recurrences and metastases, appeared to have no beneficial effect. It is not possible to assess the effects of age on prognosis in such a series, but of the 2 patients over 70 years, case 22, aged 83, died 9 years later without evidence of recurrence, and case 5, aged 73, died 2 years later, apparently free from tumour. The younger patients, cases 7 and 18 have presented too recently for comment.

There is no correlation between size and survival, but the 2 cases (10 and 11) who presented with a long history, 19 and 35 years, are alive 6 and 8 years later. Treves and Sunderland found that 61·1% of their malignant cystosarcomas phyllodes had not borne children and none of the metastasising cases had had a full term pregnancy. None of the 6 patients in groups I and II (of the present series) in which the parity state was recorded, had borne children, and 3 of them died with sarcomatosis. In group III however, 2 had borne children, and one had not.

The histological assessment of malignancy appears to be difficult. The presence of tumour giant cells does not necessarily indicate a poor prognosis. Three of the 4 tumours containing osteoid and osteoclast-like giant cells died within 19 months and the fourth is alive and well only 12 months after mastectomy. Rottino and Howley (1945) in their review of the literature, found that 16 of the 25 women with osteoid sarcomas of breast had died within 12 months.

Case 2, the only case in which invasion of blood vessels was seen (Fig. 12), presented with a sarcoma of the opposite breast 3 months later and died 9 months after being first seen. Both the patients with possibly sarcomatous tumours placed in group IV, are alive and well, but cases have been reported that were considered to be histologically benign and later presented with metastases (Lester and Stout, 1954; Collins and Dodge, 1959). This further stresses the importance of examining many blocks from fibroadenomas histologically, particularly in tumours presenting after the menopause.

### SUMMARY

The clinical and pathological findings in 23 sarcomas of breast and 2 fibroadenomas with possible sarcomatous change, are presented. All the tumours were of spindle cell type and 4 tumours contained osteoid and giant cells resembling osteoclasts. The classification used is based on their origin from benign fibroadenomas and the terms cystosarcoma phyllodes and adenofibrosarcoma are avoided.

The follow-up data suggest that this tumour has a worse prognosis than previously considered and that fibroadenomas presenting after the patient's menopause should be closely examined for evidence of malignancy.

I wish to acknowledge the help and co-operation of Dr. S. C. Dyke, Curator of the Regional Histological Collection, and the pathologists of the Birmingham Regional Hospital Board, who have kindly allowed me access to their material. I also wish to thank Dr. J. A. H. Waterhouse and Miss D. J. Powell, of the Birmingham Regional Cancer Registry.

### REFERENCES

Ariel, L.—(1961) *A.M.A. Archs Surg.*, **82**, 275.

Collins, P. G. and Dodge, O. E.—(1959) *Br. J. Surg.*, **47**, 37.

Cotchin, E.—(1958) *J. Comp. Path. Ther.*, **68**, 1.

Curran, R. C. and Dodge, O. E.—(1962) *J. clin. Path.*, **15**, 1.

D'Aunoy, R. and Wright, R. W.—(1930) *Ann. Surg.*, **92**, 1059.

Hill, R. P. and Stout, A. P.—(1942) *Archs Surg.*, **44**, 723.

Illingworth, C. F. W. and Dick, B. M.—(1941) In ' A Textbook of Surgical Pathology ', 4th edition. London (Churchill), p. 382.

Lester, J. and Stout, A. P.—(1954) *Cancer, N.Y.*, **7**, 335.

Müller, J.—(1938) ' Ueber den feinern Bau und die Formen der Krankhaften ', Berlin (Geschwulste), p. 56.

Notley, R. G. and Griffiths, H. J.—(1965) *Br. J. Surg.*, **52**, 360.

Pearse, A. G. E.—(1960) In ' Histochemistry Theoretical and Applied ', 2nd edition. London (Churchill), pp. 868, 881 and 888.

PEPLER, W. J.—(1958) *J. Path. Bact.*, **76**, 505.
ROGERS, H. AND FLO, S.—(1942) *New Engl. J. Med.*, **226**, 841.
ROTTINO, A. AND HOWLEY, C. P.—(1945) *Archs Path.*, **40**, 44.
STEWART, F. W.—(1950) In ' Atlas of Tumour Pathology. Tumours of the Breast '. Sect. IX, Fascile 34, Washington, D.C. (Armed Forces Institute of Pathology), p. 86.
TREVES, N. AND SUNDERLAND, D. A.—(1951) *Cancer, N.Y.*, **4**, 1286.
WILLIS, R. A.—(1960*a*) In ' Pathology of Tumours ', 3rd edition. London (Butterworth), pp. 210, 216, 218. —(1960*b*) In ' The Borderland of Embryology and Pathology ', 2nd edition. London (Butterworth), p. 566.